# The effectiveness of dance interventions on sleep quality: a systematic review and meta-analysis

**DOI:** 10.3389/fpubh.2026.1776902

**Published:** 2026-03-10

**Authors:** Ziqian Li, Wang Song, Yuehui Long, Yutao Zhou, Gulandanmu Ma

**Affiliations:** 1 School of Economics and Management, Shanghai University of Sport, Shanghai, China; 2 Martial Art and Arts Academy, Nanjing Sports Institute, Nanjing, Jiangsu, China; 3 School of Physical Education and Health, Wenzhou Business College, Wenzhou, Zhejiang, China; 4 School of Art, Shanghai University of Sport, Shanghai, China; 5 College of Physical Education, Hunan University of Technology, Zhuzhou, Hunan, China; 6 School of Human Sciences, University of Derby, Derby, United Kingdom

**Keywords:** adults, dance, physical activity, Pittsburgh Sleep Quality Index, sleep quality

## Abstract

**Background:**

Sleep health is increasingly recognized as central to physical and mental wellbeing, yet sleep disturbances are common and may be worsened by modern lifestyles. The WHO recognizes dance as a feasible form of physical activity, but evidence for sleep benefits is limited. This meta-analysis quantified the overall and domain-specific effects of dance interventions on sleep quality.

**Methods:**

A systematic search strategy integrating Medical Subject Headings terms and free-text keywords was employed to identify empirical studies investigating the effects of dance interventions on sleep quality across four major English-language databases. Risk of bias was assessed using the Cochrane RoB 2 tool. A meta-analysis was conducted with R 4.5.1 and Stata 18.0, pooling standardized mean differences (SMDs) under a random-effects model. Domain-specific, sensitivity, and subgroup and meta-regression analyses were performed, and publication bias was examined using Begg’s and Egger’s tests.

**Results:**

10 RCTs (711 participants) were included. Compared with controls, dance significantly improved sleep quality (SMD = 0.48, 95% CI: 0.14–0.82), representing a small-to-moderate effect. At the domain level, only subjective sleep quality remained significant after Holm multiple adjustment, with an effect size of SMD = 0.47. In the sensitivity analysis restricted to studies using the PSQI only, the effect size was attenuated (SMD = 0.355), while heterogeneity decreased substantially (*I*^2^ = 21.7%). No publication bias was detected, and the sensitivity and meta-regression analyses supported the robustness of the findings.

**Conclusion:**

Sleep is an important determinant of individual physical and mental health. Dance interventions, as a nonpharmacological approach, produce a statistically significant overall improvement in sleep quality, with a small-to-approaching-moderate effect that is supported by consistent findings across sensitivity and meta-regression analyses, and with a particularly pronounced improvement in the subjective sleep quality domain. Exploratory subgroup patterns suggested that a group-based aerobic dance program conducted three times per week, 60 min per session, over 12–16 weeks may be associated with greater benefits compared with lower-frequency or shorter-duration dance interventions. In practice, priority should be given to using homogeneous instruments and to standardized reporting of prescription components and adherence, in order to enhance the translational value of the public health evidence.

**Systematic review registration:**

PROSPERO, CRD420251104782, available from https://www.crd.york.ac.uk/PROSPERO/view/CRD420251104782.

## Introduction

Sleep quality is a comprehensive indicator used to evaluate whether an individual’s sleep is restorative, stable, and subjectively satisfactory. It encompasses multiple dimensions, including sleep latency, sleep maintenance, sleep depth, and subjective feelings upon awakening (1). At present, the Pittsburgh Sleep Quality Index (PSQI)—the most commonly used instrument in clinical and research settings—characterizes sleep status through seven domains and a global score. A global score greater than 5 is typically classified as poor sleep quality, providing a standardized framework for assessing the effects of various lifestyle interventions on sleep quality (2). Sleep health has risen to become a key problem in public health and economic governance. Epidemiological evidence and health-economic models indicate that insufficient sleep and insomnia symptoms are widespread among adults, resulting in substantial economic losses and healthcare burdens. Using five OECD countries—the United States, the United Kingdom, Japan, Germany, and Canada—as examples, work absenteeism and presenteeism attributable to insufficient sleep account for approximately 0.85–2.92% of annual GDP losses (3), and long-term imbalance in sleep duration and circadian rhythm may increase the risk of all-cause mortality by 14–34% (4). The China Sleep Quality Survey Report (2019) reported that more than 80% of the population is frequently troubled by sleep problems. Epidemiological studies further suggest that the prevalence of insomnia is approximately 27% among adults worldwide, reaching 38.2% in China—significantly higher than the global average (5). Against this backdrop, nonpharmacological strategies to optimize sleep—particularly sustainable, long-term lifestyle interventions—have become a shared focus of public health and clinical practice.

Physical activity is widely regarded as an important nonpharmacological approach to improving sleep quality (6). Multiple systematic reviews and meta-analyses have confirmed that moderate-intensity exercise has positive effects on sleep latency, sleep duration, and subjective sleep quality (7–10). The World Health Organization, in the WHO Guidelines on Physical Activity and Sedentary Behaviour (2020), explicitly lists “dancing” as a form of recreational physical activity that incorporates both aerobic and balance components, emphasizing that enjoyable and sustainable physical activity should be promoted to enhance physical and mental health (11). Beyond sleep-related outcomes, dance interventions have also been reported to benefit multiple physiological and psychological domains, including balance, gait, cognitive function, depression, anxiety, and pain-related outcomes. Compared with traditional activities such as walking and running, dance has distinctive advantages in rhythm, choreography, music, and social interaction. These features are more likely to enhance participation motivation and long-term adherence. As a form of physical activity that integrates aesthetic experience with physiological load, dance interventions have entered the global public health agenda, providing an important policy context and practical foundation for exploring pathways through which dance may improve sleep quality. However, evidence regarding the effects of dance interventions on sleep outcomes remains fragmented and is typically discussed within broader meta-analyses of exercise interventions for sleep. When dance is combined with highly heterogeneous forms of physical activity, its distinctive characteristics such as rhythm, music, choreography, and social interaction are often not evaluated independently. This limits the interpretability of the existing evidence and reduces its value for translation into targeted sleep promotion strategies. To address this limitation, the present review synthesizes randomized controlled trials of dance interventions in adults and examines sleep outcomes primarily within the PSQI framework, while also conducting exploratory comparisons across key intervention characteristics.

Accordingly, this study will conduct a systematic review and meta-analysis in accordance with the PRISMA guidelines to comprehensively evaluate the overall and domain-specific effects of dance interventions on adult sleep quality, and to examine the potential moderating roles of dance program design components and population characteristics. The specific objectives are as follows: (i) to quantify the effect size of dance interventions on overall sleep quality indicators such as the PSQI global score, and to determine the overall effectiveness of dance as a nonpharmacological sleep intervention; (ii) to conduct PSQI domain-specific analyses to examine the differential effects of dance interventions on subjective sleep quality, sleep latency, sleep duration, habitual sleep efficiency, sleep disturbances, use of sleep medication, and daytime dysfunction, thereby identifying domains that may benefit preferentially; and (iii) to use subgroup analyses and meta-regression to preliminarily test the potential moderating roles of population characteristics and intervention parameters—such as intervention frequency, session duration, intervention period, and dance type—so as to provide quantitative clues for answering which types of dance, and at what dose, are most conducive to improving sleep quality. By systematically integrating the available evidence, this study aims to provide a more robust empirical basis—with stronger evidence and clearer prescription details—for dance interventions as a nonpharmacological strategy to improve sleep quality, and to inform policy development and practical implementation regarding dance-based physical activity and sleep promotion in the public health field.

## Materials and methods

This study follows the PRISMA 2020 guidelines and has been approved and registered on the International Prospective Register of Systematic Reviews (PROSPERO), with the registration number CRD420251104782.

### Literature search strategy

On September 23, 2025, the investigators searched four databases—Web of Science, PubMed, Cochrane, and Embase—from database inception to the search date, without applying restrictions on study design or blinding at the search stage. The keyword strategy was developed by combining Medical Subject Headings terms and free-text keywords, and was not restricted to a specific sleep instrument, allowing identification of studies reporting sleep quality or related sleep outcomes assessed using validated sleep or health-related quality-of-life measures. Within each keyword set, terms were combined with OR, and the two keyword sets were combined with AND using Boolean operators. The first keyword set (“dance”) included: Dancing, Dance, Ballet, “Jazz Dance,” “Tap Dance,” “Modern Dance,” “Hip-Hop Dance,” “Hip Hop Dance,” “Line Dancing,” “Salsa Dancing,” “Square Dance.” The second keyword set (“sleep quality”) included: “Sleep Quality,” “sleep quality,” “quality of sleep.” Taking Web of Science as an example, the search strategy was: (TS = (Dancing) OR [Dancing OR Dance OR Ballet OR “Jazz Dance” OR “Tap Dance” OR “Modern Dance” OR “Hip-Hop Dance” OR “Hip Hop Dance” OR “Line Dancing” OR “Salsa Dancing” OR “Square Dance”]) AND (TS = (Sleep Quality) OR [“Sleep Quality” OR “sleep quality” OR “quality of sleep”]).

### Inclusion and exclusion criteria

All included studies were empirical investigations selected according to the evidence-based medicine PICOS framework, namely: Participants, Intervention, Comparison, Outcome, and Study design (12). After merging the records retrieved from the four databases and importing them into EndNote, duplicate publications were removed. After confirming the inclusion and exclusion criteria, the first author screened titles and abstracts and then conducted full-text screening, while another author performed an independent assessment to determine eligibility. In cases of disagreement, a third author was consulted or consensus was reached through discussion within the research team. After all studies were reviewed and discussed, the final set of included studies was determined. The detailed inclusion and exclusion criteria are presented in Table 1. In summary, we included randomized controlled trials (RCTs) and non-randomized comparative studies that evaluated a structured dance intervention (of any style) against a control condition (e.g., wait-list, usual care, or an active control) in adult populations, with sleep quality or related parameters as a primary or secondary outcome. Studies were excluded if they: (i) did not involve a structured dance program as the core intervention (e.g., studies focused solely on general exercise, sports, or non-movement-based therapies); (ii) examined non-structured dance activities (e.g., free-form social dancing without a defined protocol); (iii) exclusively enrolled regular or professional dancers.

**Table 1:** Inclusion and exclusion criteria.

PICOS	Inclusion criteria	Exclusion criteria
Participants	Adults (≥18 years)	<18 years; regular exercisers
Intervention	Structured dance	Non-dance Unstructured dance
Comparison	Non-exercise controls	No control group
Outcome	Subjective scales	Insufficient data
Study design	RCTs	Conference abstracts Thesis reviews Case reports

### Quality assessment of the included studies

All included studies were randomized controlled trials (RCTs) that met the predefined PICOS eligibility criteria, and no eligible RCTs were excluded on the basis of methodological quality. Risk of bias was assessed using the Cochrane RoB 2 tool to characterize internal validity and inform interpretation of the findings, rather than as a study exclusion criterion. RoB 2 evaluates the risk of bias across five domains: the randomization process, deviations from intended interventions, missing outcome data, measurement of the outcome, and selection of the reported result. In accordance with Cochrane guidance, each domain was judged as “low risk,” “some concerns,” or “high risk” based on the RoB 2 criteria (13). If the two reviewers reached different judgments for any domain or for the overall risk-of-bias assessment during independent evaluation, the discrepancy was resolved through discussion. If disagreement persisted, a third reviewer adjudicated until consensus was achieved.

### Statistical analysis

Screening and data extraction were conducted independently by the first and second authors. If data were missing or presented only in graphical form, the corresponding authors were first contacted to request the required information. If no response was received within 2 weeks, WebPlotDigitizer 4.1 was used to extract data from figures. Studies for which the missing information could not be obtained were excluded. Participants’ age was calculated using the mean values reported in the original studies; if only an age range was provided, the median was used. For studies reporting only the standard error (SE), values were converted using the formula SD = SE × $\sqrt{n}$ to ensure that calculations were based on a consistent standard deviation (SD) (14).

This study used R 4.5.1 (the metafor package) and Stata 18.0 to calculate the standardized mean difference (SMD) as the effect size for the impact of dance interventions on sleep quality. The clinical interpretation of effect sizes followed Cohen’s conventions: SMD < 0.2 indicates a trivial effect, 0.2 ≤ SMD < 0.5 a small effect, 0.5 ≤ SMD < 0.8 a moderate effect, and SMD ≥ 0.8 a large effect (15). Heterogeneity was assessed using Cochran’s *Q* test and the *I*^2^ statistic. A prespecified decision rule was applied for model selection: when heterogeneity was low (*I*^2^ ≤ 50%), a fixed-effect model was used; when heterogeneity was substantial (*I*^2^ > 50%), a random-effects model was used. This rule was used to align model assumptions with the observed degree of between-study variability, given the potential clinical and methodological differences across included studies. Publication bias was evaluated using funnel plots and Begg’s and Egger’s tests. Statistical significance was defined as *p* < 0.05.

To explore potential sources of heterogeneity, the following analyses were conducted. (i) Subgroup analyses: single-variable mixed-effects models were constructed according to population, intervention frequency, dance type, and intervention period. Moderation was evaluated using the between-group heterogeneity statistic QM (moderator test). Holm multiple adjustment was applied to the *p* values from the four subgroup analyses, and statistical significance was determined using P(Holm). To assess the extent to which each variable explained heterogeneity, heterogeneity estimates from the baseline model without moderators were compared with those from models including each moderator; residual *τ*^2^, residual *I*^2^, and pseudo-*R*^2^ were reported to quantify the proportion of heterogeneity explained. (ii) Sensitivity analyses: these included leave-one-out analyses and checks for instrument consistency. (iii) Meta-regression: single-variable meta-regression was performed with instrument type (PSQI vs. non-PSQI) as a categorical moderator. QM (moderator test) and its *p* value were reported, along with post-regression residual heterogeneity statistics (*Q*, *τ*^2^, and *I*^2^).

## Results

### Search results

**Figure 1:**
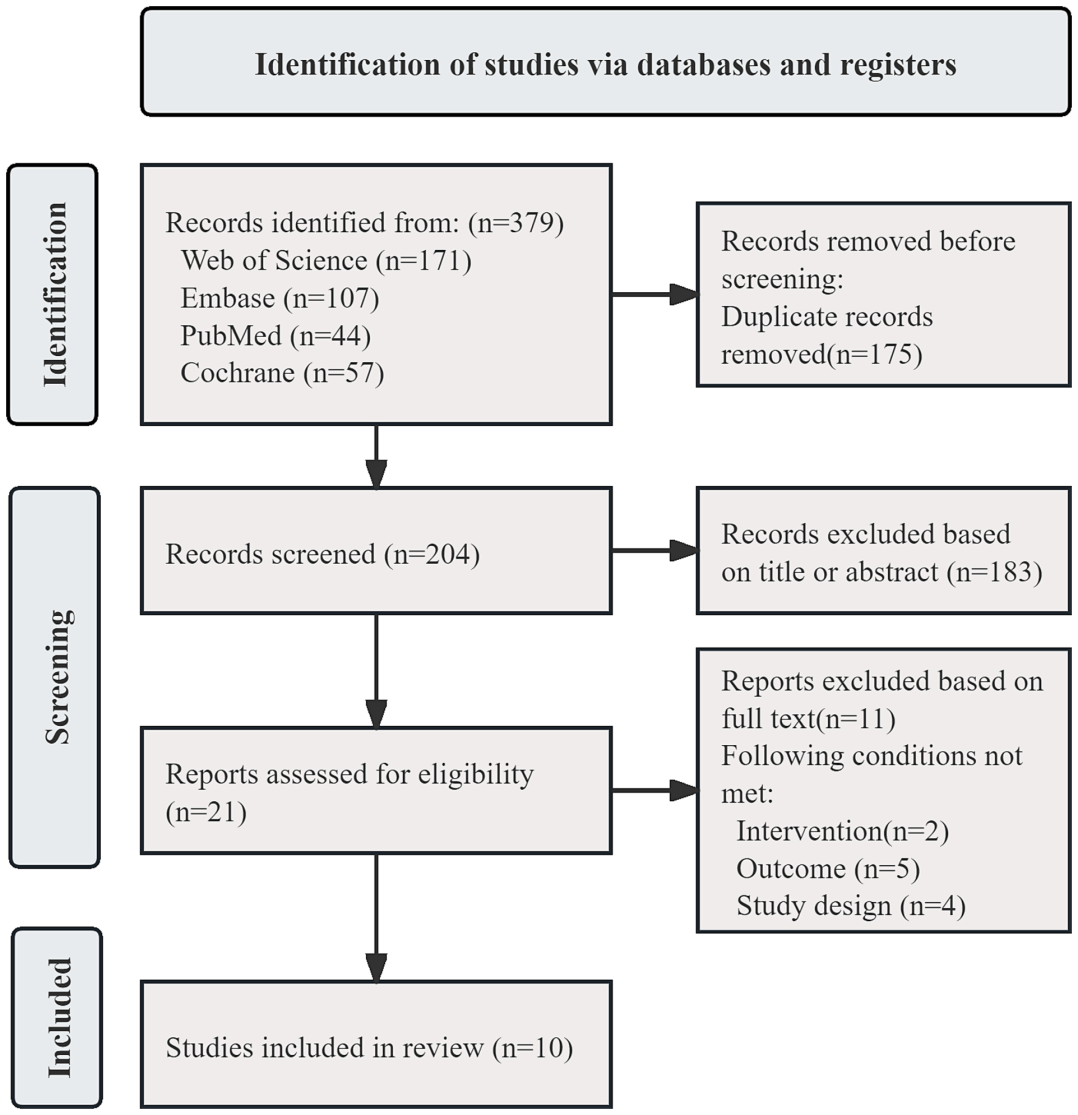
Flowchart of literature screening.

As shown in Figure 1, a total of 379 records were identified through the search strategy. After removing duplicates in EndNote, 204 records remained. Following title and abstract screening, 21 articles were retained for full-text review. After full-text assessment in strict accordance with the inclusion and exclusion criteria, 10 studies were ultimately included (16–25).

### Description of included studies

A total of 10 randomized controlled trials (RCTs) from multiple countries were ultimately included. Seven studies enrolled women only (16–18, 21, 23–25), and three studies included mixed-sex samples (19, 20, 22). The duration of each intervention session ranged from 50 to 90 min, with 60 min being the most common. Intervention frequency varied from once to three times per week. The most common frequency was twice per week in five studies (16–18, 20, 24), followed by three times per week in four studies (19, 21, 23, 25). Intervention periods ranged from 3 to 16 weeks, with 16 weeks being the most common in five studies (17–19, 21, 25). All interventions were delivered as group-based classes. Control conditions typically included wait-list control, health education, or usual activities; one study used compression stockings. Outcomes were assessed primarily using the PSQI, with one study reporting FPS (25). A summary of participants’ characteristics and analysis of the studies included in the meta-analysis are presented in Table 2.

**Table 2:** Basic characteristics of the included studies.

Study ID and year	Category	*n*	Gender		Age	Intervention design		Duration frequency period	Outcome	Adverse events
			M	F	Mean ± SD	EG	CG			
Min et al. (2025) (16)	Group dance	E = 14 C = 13	0	27	32.81 ± 5.71	Gyrokinesis	Compression stock	60 min 2/week 8 weeks	PSQI	Not reported
Bocchi Martins et al. (2025) (17)	Group dance	E = 23 C = 24	0	47	53.41 ± 2.8	Jazz	Wait list	60 min 2/week 16 weeks	PSQI	Not reported
Fausto et al. (2025) (18)	Group dance	E = 23 C = 24	0	47	53.19 ± 3.39	Jazz	Wait list	60 min 2/week 16 weeks	PSQI	Not reported
Song et al. (2024) (19)	Group dance	E = 45 C = 44	24	71	75.97 ± 6.31	Aerobic dance	Health education	60 min 3/week 16 weeks	PSQI	Not reported
Sánchez-Alcalá et al. (2024) (20)	Group dance	E = 47 C = 45	34	58	71.83 ± 2.96	Aerobic dance	Wait list	60 min 2/week 12 weeks	PSQI	Not reported
Boing et al. (2023) (21)	Group dance	E = 25 C = 24	0	49	55.37 ± 10.43	Belly dance	Health education	60 min 3/week 16 weeks	PSQI	Not reported
Mar Lopez-Rodriguez et al. (2017) (22)	Group dance	E = 53 C = 42	24	71	22.33 ± 4.12	Bio-dance	Wait list	90 min 1/week 4 weeks	PSQI	Not reported
Serrano-Guzmán et al. (2016) (23)	Group dance	E = 35 C = 32	0	67	69.07 ± 4.41	DMT	Routine	50 min 3/week 8 weeks	PSQI	Not reported
Ho et al. (2016) (24)	Group dance	E = 69 C = 70	0	139	48.9 ± 8.2	DMT	Routine	90 min 2/week 3 weeks	PSQI	Not reported
Tchae-Won et al. (2011) (25)	Group dance	E = 29 C = 30	0	59	69.5 ± 6.3	Aerobic dance	Wait list	60 min 3/week 16 weeks	FPS	Not reported

E = experiment group; C = control group; M = mean; SD = standard deviation; DMT = dance movement therapy; PSQI = Pittsburgh Sleep Quality Index; FPS = faces pain scale.

### Assessment of risk of bias

**Figure 2:**
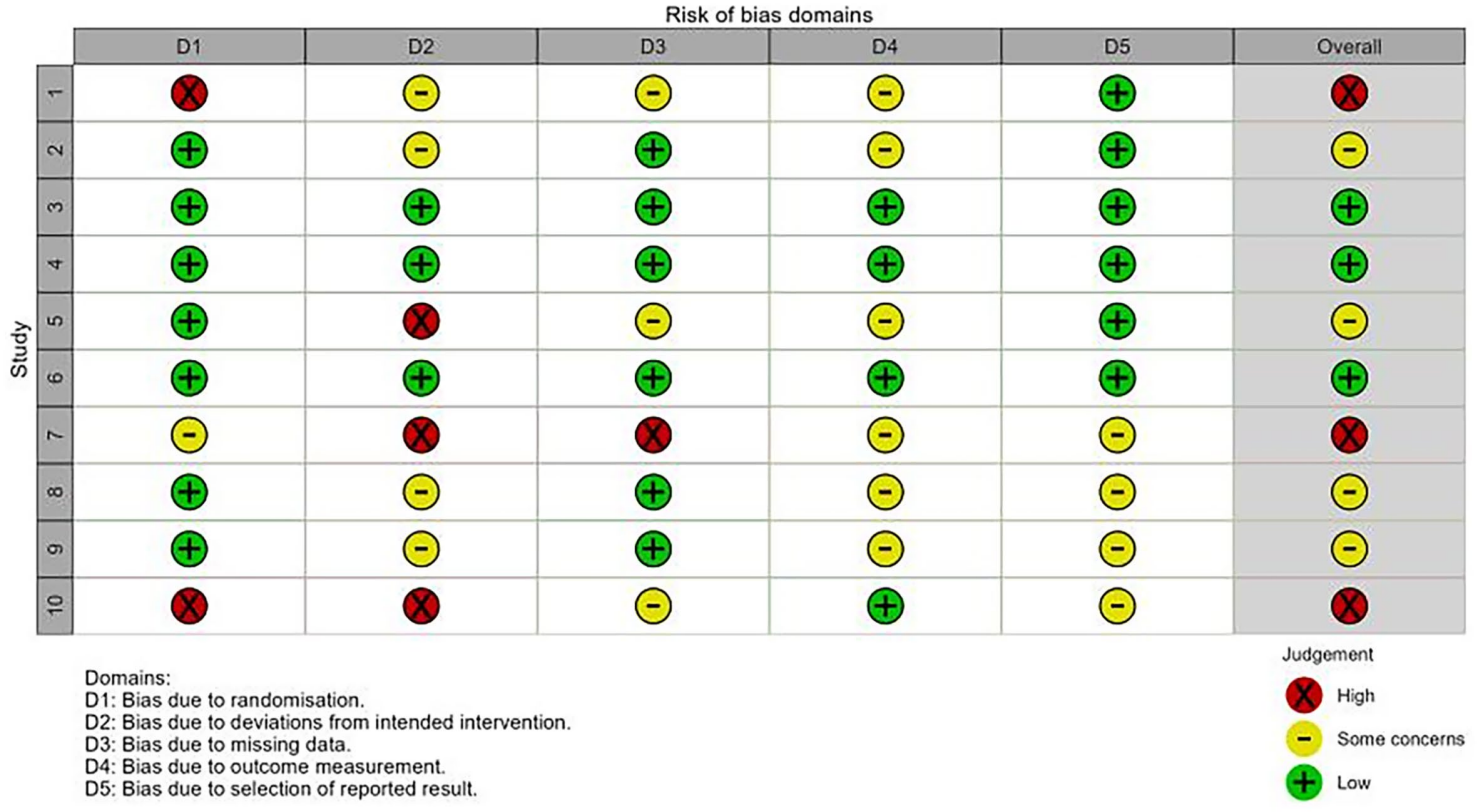
Quality assessment of literature.

As shown in Figure 2, across the 10 included RCTs, the overall risk of bias was judged as high risk (30%) (16, 22, 25), some concerns (40%) (17, 20, 23, 24), and low risk (30%) (18, 19, 21). The primary reasons were: (i) baseline imbalance; (ii) lack of assessor blinding when administering subjective instruments; and (iii) differential loss to follow-up or unclear handling of missing data. Because blinding is difficult to implement in dance interventions, this was not treated as a mandatory criterion. Overall, there remains room for improvement in the transparency of allocation concealment and blinding of outcome assessment, whereas risks related to handling missing data and selective reporting were considered manageable.

### Pooled effect-size analysis of dance interventions on sleep quality

To facilitate interpretation, the direction of effects was standardized such that positive values favor the intervention. Under this convention, a positive SMD indicates a lower PSQI global score, and because lower PSQI scores reflect better sleep, this corresponds to improved sleep quality.

### Primary outcome analysis of dance interventions on sleep quality

**Figure 3:**
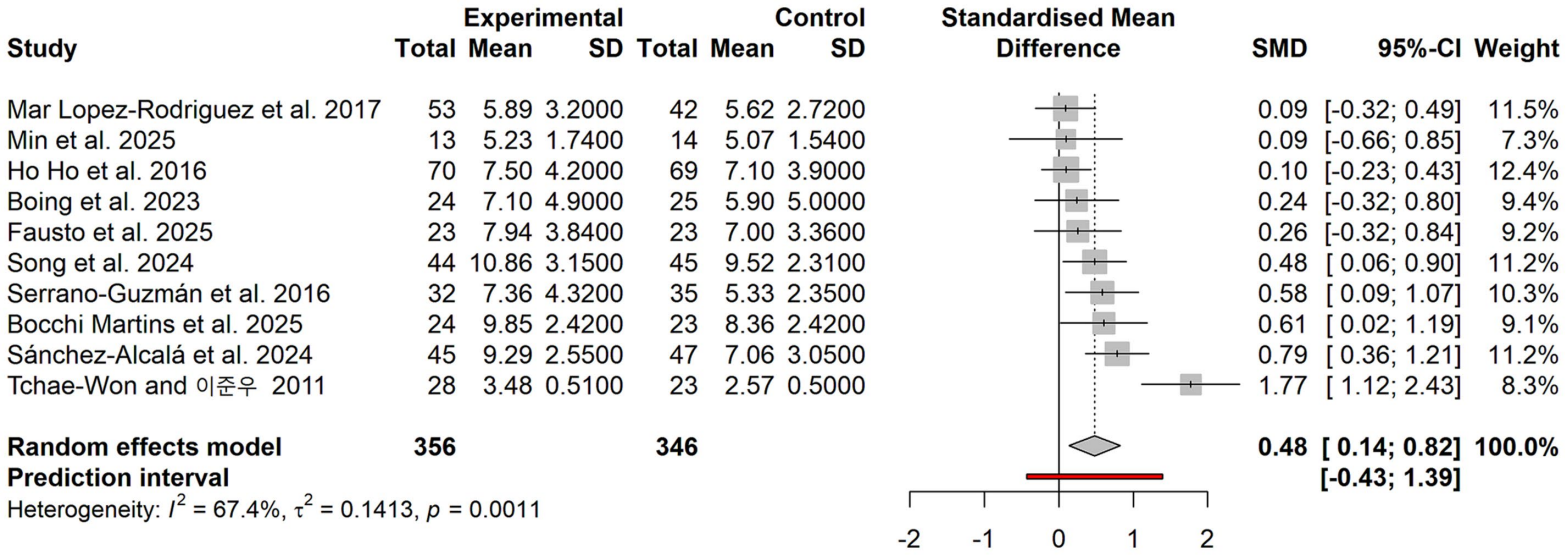
Forest plot of the overall effect of dance intervention on improving sleep quality.

As shown in Figure 3. When all outcome measures were included, the pooled effect size was SMD = 0.48 (95% CI: 0.14–0.82), with *I*^2^ = 67.4% and *p* = 0.001. Compared with the control group, the dance intervention group showed a statistically significant improvement in sleep quality, with an effect size that was small to approaching moderate.

### Domain-specific outcome analysis of dance interventions on sleep quality

**Figure 4:**
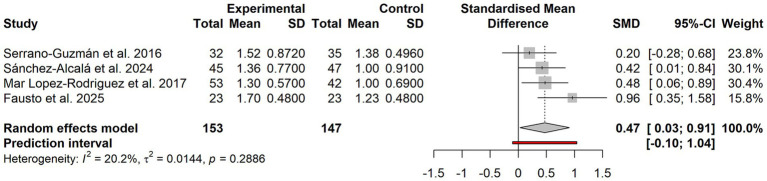
Forest plot of domain-specific effects on subjective sleep quality with dance interventions.

As shown in Figure 4. Among the 10 included studies, only four reported the full set of outcomes for all seven PSQI domains in sufficient detail (18, 20, 22, 23). The remaining six studies reported only the overall outcome and selected domain outcomes; therefore, the number of studies contributing to each domain-specific analysis varied. The domain-specific analyses showed that only the subjective sleep quality domain demonstrated a significant intervention effect (SMD = 0.47, 95% CI: 0.03–0.91; *I*^2^ = 20.2%) and remained significant after Holm multiple adjustment (P(Holm) < 0.001). The other six domains—sleep latency, sleep duration, habitual sleep efficiency, sleep disturbances, use of sleep medication, and daytime dysfunction—were not statistically significant after Holm adjustment.

### Subgroup analysis results

Given the relatively high overall heterogeneity, further subgroup analyses were performed to examine potential moderators and identify sources of heterogeneity (26). Single-factor subgroup analyses were conducted for four variables—population, intervention frequency, dance type, and intervention period. After Holm multiple adjustment, none of the between-group differences (QM) reached statistical significance. Specifically, for population: QM = 1.39, Pm = 0.709, Pm(Holm) = 0.709; for intervention frequency: QM = 1.97, Pm = 0.160, Pm(Holm) = 0.480; for dance type: QM = 6.33, Pm = 0.0967, Pm(Holm) = 0.387; and for intervention period: QM = 1.14, Pm = 0.285, Pm(Holm) = 0.569.

Compared with the main-effect model (*τ*^2^₀ = 0.141, *I*^2^₀ = 67.4%), the subgroup analyses yielded the following explanatory power (pseudo-*R*^2^): the population variable showed −35.7% (*τ*^2^ = 0.171, *I*^2^* = 71.3%), the intervention frequency variable explained 8.97% (*τ*^2^* = 0.115, *I*^2^* = 64.2%), the dance type variable explained 31.1% (*τ*^2^* = 0.087, *I*^2^* = 55.9%), and the intervention period variable showed essentially no change. These findings indicate that, given the current sample structure, the subgroups had limited explanatory power and may even have amplified residual heterogeneity. Based on the non-significant between-group differences and the limited explanatory power (pseudo-*R*^2^), none of the four candidate variables can be confirmed as an effective moderator. However, the dance type subgroup showed a consistent trend toward reducing heterogeneity, suggesting that the choice of dance modality may provide some explanatory value for the observed effects.

The pooled effect sizes across subgroups showed a trend toward larger SMDs at higher intervention frequencies and longer intervention periods; however, overall within-subgroup heterogeneity remained high, suggesting that residual heterogeneity may be driven by implementation differences such as dose parameters and intervention delivery quality. Based on the pooled effect sizes and confidence intervals, dance interventions demonstrated a moderate effect in populations with cognitive impairment (SMD = 0.618); intervention frequency of more than twice per week showed a moderate-to-large effect (SMD = 0.737); aerobic dance achieved a large effect (SMD = 0.966); jazz dance showed a small-to-moderate effect (SMD = 0.429); and an intervention period of more than 8 weeks demonstrated a moderate effect (SMD = 0.648). Given that the overall between-group differences were not statistically significant, these findings are presented as trend-level descriptions only and should not be interpreted as evidence of statistically significant moderation. Details are provided in Table 3.

**Table 3:** Subgroup analysis results on the effects of dance intervention on sleep quality.

Moderator	Subgroup	*K*	SMD (Hedges’ *g*)	95%CI	*I* ^2^	*τ*^2^*	*I*^2^*	Pseudo-*R*^2^	pm	pm (Holm)
Population	Health adults	3	0.646	−0.436, 1.728	89%	0.172	71.3%	−35.7%	0.709	0.709
	Postmenopausal women	2	0.429	0.008, 0.85	0%					
	MCI	2	0.618	0.358, 0.878	0%					
	Cancer patient	2	0.289	0.155, 0.423	0%					
Frequency	≤2 sessions	6	0.318	0.055, 0.581	42%	0.115	64.2%	8.97%	0.16	0.48
	>2 sessions	4	0.737	0.167, 1.307	78%					
Type	Jazz dance	2	0.429	0.008, 0.85	0%	0.087	55.9%	31.1%	0.096	0.387
	Aerobic dance	3	0.966	0.315, 1.616	81%					
	DMT	2	0.298	−0.164, 0.761	58%					
	Multimodal dance	3	0.132	−0.172, 0.438	0%					
Duration	<8 weeks	5	0.334	0.030, 0.639	54%	0.128	66.5%	−0.963%	0.285	0.569
	>8 weeks	5	0.648	0.159, 1.138	74%					

*K* = the number of included studies; *I*^2^ = heterogeneity within each subgroup level; *I*^2^* = heterogeneity between subgroups; Pm = the *p* value for between-group differences; MCI = mild cognitive impairment; DMT = dance movement therapy.

### Sensitivity analyses

Compared with the primary analysis including all studies, restricting the analysis to studies that used the PSQI as the outcome measure reduced heterogeneity to *I*^2^ = 21.7%, indicating that the findings were more stable and that overall heterogeneity decreased substantially when a homogeneous instrument was used. The leave-one-out analysis showed that although certain studies exerted some leverage on the overall estimate, the direction of the pooled effect remained consistent after removing any single study. This suggests that the overall results are robust, with no evidence that a single study dominated the observed trend; therefore, the main conclusions were not materially affected. The results of the sensitivity analyses are shown in Table 4.

**Table 4:** Sensitivity analyses of pooled effects.

Category	*K*	Hedges’ *g*	95%CI	*τ* ^2^	*I* ^2^	*Q*	*P*_(*Q*)_
Non-PSQI	10	0.48	0.20, 0.75	0.127	66.7%	27.04	0.001
PSQI	9	0.36	0.17, 0.54	0.016	21.7%	10.21	0.25

### Results of the meta-regression analyses

Instrument type (PSQI vs. FPS) was entered as a categorical moderator in the meta-regression. The results indicated a significant between-group difference (QM = 14.338, *p* = 0.00015). Compared with PSQI, the effect size associated with FPS was significantly larger, with an estimated coefficient increase of approximately 1.42 (SE = 0.37, *p* = 0.00015). Given that FPS was reported in only one study, this meta-regression is presented solely as an exploratory analysis intended to help explain heterogeneity and should not be interpreted as confirmatory evidence that differences in effect estimates are attributable to the type of outcome instrument used. These findings do not alter the primary conclusions derived from analyses restricted to PSQI.

### Publication bias assessment

**Figure 5:**
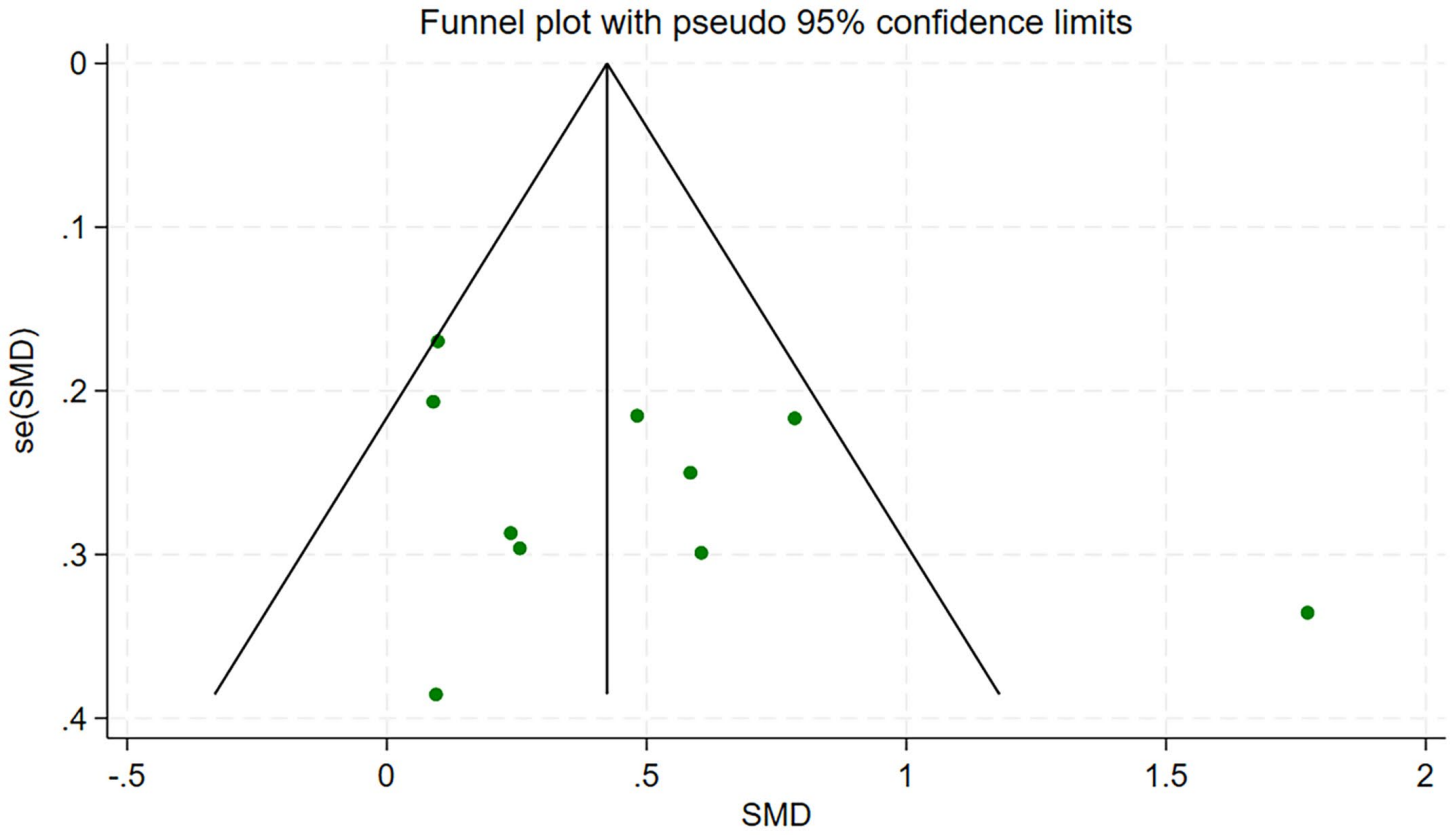
Funnel plot of included studies.

As shown in Figure 5. The funnel plot showed that the scatter points were relatively symmetric around the vertical line representing the pooled effect, with no obvious “missing corner.” Overall, there was no statistical evidence of publication bias among the 10 included studies. Begg’s test yielded *p* = 0.245 (after continuity correction, P(Holm) = 0.283), and Egger’s test yielded *p* = 0.237, indicating a low likelihood of publication bias in the included literature.

## Discussion

### Reappraising the findings: from “dance more, sleep better” to “a limited but robust improvement”

In public and policy discourse, the idea that more exercise—or more dancing—leads to better sleep is often treated as common sense. However, the pooled evidence from this study, synthesizing 10 RCTs with 711 participants, indicates that dance interventions yield a small-to-approaching-moderate improvement in overall sleep quality (SMD = 0.48). The improvement is concentrated primarily in the subjective sleep quality domain (SMD = 0.47), whereas other PSQI domains—many of which are more objective in nature—did not remain statistically significant after Holm multiple adjustment.

When the analysis was restricted to studies using the PSQI only, the pooled effect attenuated slightly (SMD = 0.36), but heterogeneity decreased markedly (*I*^2^ = 21.7%). This suggests that, under conditions of instrument consistency, the impact of dance on sleep is not a “strong-acting remedy,” but rather a methodologically robust and reproducible intervention with a small-to-moderate effect. These results challenge the simplistic assumption that dance improves every dimension of sleep in a linear manner, shifting interpretation toward a more cautious conclusion: dance appears to primarily change individuals’ overall subjective experience of sleep, rather than simultaneously and substantially reshaping multiple components of sleep structure.

### Evidence landscape and the meaning of heterogeneity: dance interventions do not affect all sleep quality domains equally

Current evidence indicates pronounced domain-specific heterogeneity in the effects of dance interventions on sleep quality. Specifically, improvement is most evident in the subjective sleep quality domain, whereas more objective PSQI domains—such as sleep latency, sleep duration, and habitual sleep efficiency—do not show consistent benefits after Holm multiple adjustment (27, 28). Subjective sleep quality is, by nature, a context-dependent integrative appraisal, encompassing retrospective judgments about sleep initiation and maintenance, daytime functional status, sleep satisfaction, and perceived controllability, among multiple dimensions (29–31). The distinctive components of dance interventions—including music synchronization, rhythmic regulation, and group interaction—may preferentially operate through psychosocial pathways such as regulating emotional tension, enhancing self-efficacy, and reinterpreting bodily signals (32–37). As a result, individuals may experience a subjective sense of “sleeping better” even before objective sleep parameters show meaningful changes. This pattern of subjective-first improvement has been repeatedly observed across populations such as those with diabetes, fibromyalgia, and cancer, suggesting cross-population stability of this effect (38). These domain-specific differences caution clinicians and community practitioners against presuming “synchronous improvements across multiple domains.” In sleep disturbance interventions, perceived restorative sleep and objectively measured sleep duration do not necessarily change in a coordinated manner (39–42). If practice guidelines overemphasize longer sleep duration or shorter sleep latency as the primary efficacy indicators, they may systematically underestimate the unique value of dance interventions in reshaping the overall sleep experience, alleviating anticipatory nocturnal anxiety, and improving daytime functioning (23, 43). Therefore, positioning subjective sleep quality as a core outcome that can be measured independently and that carries clinical significance better aligns with the strengths and theoretical mechanisms of dance interventions, and is also consistent with the current clinical trial trend emphasizing patient-reported outcomes (PROs).

### Context and fit: the potential of dance as a “sleep prescription”

Although the subgroup analyses did not identify statistically significant moderation effects (population type, intervention frequency, dance type, and intervention period all showed P(Holm) > 0.05), the distribution of effect sizes still reveals meaningful contextual features and directions for optimization. At the population level, subgroups of postmenopausal women and older adults with mild cognitive impairment showed moderate effect sizes, whereas the effect among patients receiving adjuvant chemotherapy for breast cancer was close to zero (17, 20, 21). The observed population differentiation suggests that dance interventions may be better suited as an adjunctive approach for managing chronic, potentially reversible sleep problems, rather than serving as a primary sleep solution under acute physiological stress or severe disease conditions. In the latter contexts, complex inflammatory burden and circadian rhythm disruption may create pathophysiological barriers that are difficult to overcome through exercise-based interventions alone (44).

The dose–response relationship appears to be nonlinear. Subgroup patterns for frequency and intervention period suggest that a group-based aerobic dance program delivered more than twice per week, 60 min per session, and for more than 8 weeks shows the most favorable trend in effect size, whereas lower-frequency programs (≤2 sessions/week) or shorter intervention periods (<8 weeks) appear to yield limited benefits (25, 38). However, heterogeneity within some high-frequency programs was excessive (*I*^2^ = 78%), suggesting that excessive load may activate the sympathetic nervous system and thereby offset the benefits of dance interventions. This phenomenon appears particularly pronounced in dancer populations, where training load has been reported to be negatively associated with sleep quality (40, 45, 46). Evidence from exercise physiology further indicates that timing and intensity management of the prescription are as critical as frequency and duration. High-intensity exercise performed in the evening—if completed within 4 h of bedtime—may significantly delay sleep onset and reduce sleep efficiency; in contrast, when scheduled at least 6 h before bedtime, negative effects may be avoided and sleep-promoting effects may even occur (47). This provides a clear caution for dance class design: evening sessions should tightly control the interval between the end of the class and an individual’s habitual bedtime to prevent autonomic imbalance driven by excessive activation from disrupting sleep homeostasis (48).

### Methodological and measurement reflections: what may have been overestimated or underestimated in prior research

Although no clear publication bias was detected in this study, suggesting that selective publication of “any statistically significant improvement” trials may be relatively well controlled, the existing evidence may still systematically deviate from the true effect in two directions from a methodological perspective. The first is measurement-structure drift: the interchangeable use of instruments such as the PSQI and FPS can contribute to outcome heterogeneity. Because only one included study reported FPS, the PSQI-based findings should be considered the primary evidence base for interpreting the effects of dance interventions on sleep quality, while FPS-related results should be treated as Supplementary material (25). Consistently, when analyses were restricted to studies using the PSQI only, the pooled effect size decreased and heterogeneity was substantially reduced. In theory, future research should make more explicit and rigorous choices regarding which sleep construct is being assessed. A second issue is the lack of detailed reporting on prescription and implementation: most studies provide only coarse information such as “sessions per week” and “minutes per session,” while offering limited detail on intensity, progression, and adherence. Objective indicators—such as heart rate, ratings of perceived exertion, or wearable-derived load metrics—are often absent, and few studies systematically document “co-intervention behaviors” in control groups. This reporting gap may lead to an underestimation of the true effect under conditions of well-managed prescriptions and high adherence, and it also makes it difficult to identify negative evidence attributable to poor implementation quality. In other words, the current body of dance-and-sleep research more often answers: “On average, how much improvement occurs when dance programs with vague intensity and variable delivery quality are used?” rather than: “What is the true effect of a reproducible dance prescription with clearly specified parameters in a defined population?” The latter is typically the information most needed for clinical and public health decision-making.

### Theoretical integration: from behavioral control to a dual-pathway framework of “affective–social” and “physiological–circadian”

Building on existing theoretical foundations, this study further attempts to embed the domain-specific findings and dose patterns into a more context-sensitive framework. Integrating evidence from neuroscience and exercise psychology, we propose that the effects of dance interventions on sleep quality may operate primarily through two interwoven pathways.

### Behavioral control perspective: closed-loop feedback and the reconstruction of perceived control

From a behavioral control perspective, dance training constitutes a feedback-dependent closed-loop system: guided by musical rhythm and visuoproprioceptive cues, participants refine their steps, timing, and spatial positioning through a cycle of perception–comparison–adjustment (49, 50). Across the 10 included trials, all interventions were delivered as group-based classes, typically as structured programs conducted 2–3 times per week, for approximately 60 min per session, over 8–16 weeks. In this process, participants not only achieved a sufficient volume of physical activity, but also accumulated a sense of bodily control and predictability through repeated cycles of “error–correction–mastery.” The domain-specific findings of this study indicate that dance interventions most consistently improved subjective sleep quality first, whereas no uniform significant effects were observed across other, more objective PSQI domains.

Importantly, subjective sleep quality reflects more than simply “whether one slept well”; it also incorporates integrative judgments such as satisfaction with sleep and whether sleep is perceived as being within one’s control. On this basis, it can be inferred that sustained closed-loop motor feedback training may help individuals rebuild trust in, and perceived control over, their bodily rhythms during the daytime, thereby reducing anticipatory pre-sleep anxiety (e.g., “I will sleep poorly again”). Such changes may manifest first in subjective experience rather than immediately reshaping each objective sleep indicator (51). This interpretation still requires validation in future studies through longitudinal measurement of psychological variables such as perceived behavioral control and bodily trust. Nonetheless, it offers a micro-level mechanistic hypothesis with explanatory value for the observed pattern in which improvements are preferentially evident in subjective sleep quality.

### “Affective–social” and “physiological–circadian” pathway

Synchronization between music and movement, group interaction, and positive reshaping of body image may help reduce anxiety and depression while enhancing self-efficacy and social connectedness (52, 53). Evidence from an individual study suggests that, in community samples, improvements in sleep associated with square dancing can be explained by a serial mediation pathway involving increased social support and reduced depressive symptoms (54). In the present sample, all interventions were delivered as group-based classes, and control conditions were typically wait-list control, health education, or usual activities. This implies that, in addition to receiving an exercise stimulus, the intervention group also gained exposure to a stable collective rhythm and social context. Accordingly, the effects of dance interventions on sleep were concentrated in the highly affect-laden domain of subjective sleep quality, consistent with prior dance research. On this basis, the affective–social pathway can be articulated as follows: dancing within a predictable group rhythm may reduce emotional tension while increasing social support and self-efficacy, thereby lowering pre-sleep cognitive and emotional load and improving subjective sleep quality. This account aligns closely with the observed small-to-moderate overall effect size and the domain-specific pattern of findings, and it provides a theoretical rationale for more systematically incorporating measures of affect and social connectedness in future experimental designs (55).

Moderate aerobic loading may promote neurotransmitter release (56) and regulate the hypothalamic–pituitary–adrenal (HPA) axis and inflammatory activity (57). When combined with a regular practice schedule, it may also function as a form of social zeitgeber (58), helping the body recalibrate circadian rhythms (59) and thereby improving sleep architecture and efficiency over a longer time scale. The subgroup patterns in this study suggest that more rhythmic and sustained aerobic dance programs—delivered more than twice per week and lasting longer than 8 weeks—tend to show moderate, and in some cases near-large, effect sizes. When the exercise dose is relatively low, subjective improvements driven by the affective–social pathway may emerge first, while deeper physiological and circadian restructuring may remain insufficient. Once frequency and intervention period reach a certain threshold, the physiological–circadian pathway may become increasingly salient; however, the limited sample size and coarse reporting of prescription parameters constrain a more fine-grained characterization of this dose–response relationship.

The dual-pathway model provides a concise conceptual summary of the patterns observed in this review, but it should not be interpreted as a mechanism tested or confirmed by the included trials. When intervention dose is lower, subjective improvements may be more apparent, whereas physiological and circadian adaptation may require sufficient frequency and duration. This temporal hypothesis should be evaluated in future adequately powered studies using longitudinal measurement and, where feasible, dose-matched comparisons, rather than being treated as a definitive interpretation of the present meta-analytic findings.

### Limitations, social context, and future directions

This study has several limitations that warrant a cautious interpretation, including a limited sample size, participant groups concentrated primarily among women and older adults, and insufficient evidence based on objective sleep indicators. All interventions were delivered as group-based classes, typically conducted in hospitals, community centers, or universities, with a lack of typical home-based self-directed programs, which may constrain the generalizability of the findings to other settings. In addition, intervention implementation was often reported in coarse terms (e.g., sessions per week and total weeks), with limited documentation of actual intensity, progression strategies, and adherence details, making it difficult to establish a precise dose–response curve.

From a sociological perspective, these limitations also reveal a “blind spot” in the current evidence base: most trials have treated dance primarily as a “nonpharmacological exercise program,” with limited measurement of its social time structure and community-relational effects. In fact, nearly all included interventions were delivered at fixed weekly time slots, drawing participants into a predictable collective rhythm over a sustained period. This process of “reconstructing a public temporal axis” may itself be an important mechanism for repairing social jetlag and buffering work–family stress, yet it has not been formally operationalized as a variable in quantitative research. Nevertheless, the findings point to three priority directions for future research.

### Expanding sample size and contextual diversity

Multicenter trials should be conducted in men, younger adults, multimorbid populations, and across diverse cultural contexts to test the feasibility and effectiveness of dance interventions under different sociocultural conditions. Intervention settings (e.g., community squares, long-term care facilities, hospital rehabilitation departments) should also be documented more systematically, in order to distinguish the moderating roles of different “social spaces” on improvements in sleep quality.

### Refining the prescription and monitoring implementation

At the design stage, studies should predefine intensity ranges, progression schedules, and adherence management strategies. Tools such as heart-rate monitoring, accelerometers, and wearable devices should be used to incorporate implementation quality into explanatory models rather than treating it as background information only. It is also recommended to measure variables such as perceived behavioral control and bodily trust to test whether the behavioral control pathway mediates the evidence chain linking dance and sleep quality.

### Multidimensional subjective and objective outcomes and long-term follow-up

Subjective scales, objective sleep measures (polysomnography or actigraphy), and affective/cognitive indicators should be collected in parallel, with extended follow-up periods to distinguish short-term experiential improvement from long-term restructuring of sleep architecture. Where feasible, measures of social support, neighborhood connectedness, and time-use structure may also be included to evaluate whether dance indirectly improves sleep by increasing “community social capital” and a sense of “collective rhythm.”

Overall, this study does not provide a simplistic answer that “dance can cure insomnia.” Instead, it presents a relatively clear yet still evolving evidence landscape, in which the certainty of evidence can be regarded as low to moderate due to heterogeneity across studies and limitations in the available data. Within this context, when implemented in appropriately selected populations with moderate exercise load and high-quality delivery, dance interventions show potential as a form of sleep promotion that could be institutionalized and prescribed. At the same time, dance also serves as a lens through which the complexity of sleep problems becomes visible, suggesting that before pursuing a “one-size-fits-all” solution, future work should prioritize identifying individual differences, social context, and cultural meaning. Only on this basis can warmer, more context-sensitive public health intervention strategies be developed.

## Conclusion

Based on pooled evidence from 10 randomized controlled trials involving 711 participants, this study concludes that dance interventions, as a nonpharmacological approach, can lead to small-to-moderate improvements in sleep quality among adults. The primary conclusions are driven by PSQI-based evidence, while findings from the single study using FPS are presented as Supplementary material. At the domain level, only subjective sleep quality remained robustly significant after Holm multiple adjustment, with an effect size close to moderate, suggesting that dance may preferentially improve individuals’ subjective sleep experience primarily through mechanisms related to social connectedness and affect regulation. Although the subgroup analyses and meta-regression did not identify definitive moderators, the distribution of effects suggests that programs delivered approximately 2–3 times per week, about 60 min per session, for 12–16 weeks, predominantly as group-based aerobic dance classes, may be associated with greater benefits. Given that between-group differences were not statistically significant, these findings should be interpreted as hypothesis-generating cues for prescription optimization rather than deterministic recommendations.

From both methodological and practical perspectives, future high-quality RCTs should more precisely specify and monitor prescription components—including frequency, intensity, session duration, progression, and adherence—while concurrently collecting subjective and objective sleep measures alongside physiological indicators. Trials should also standardize stratification and reporting of baseline characteristics and control conditions to enhance generalizability and translational value. At the implementation level, priority should be given to using homogeneous instruments (e.g., the PSQI) and to standardized reporting of prescription parameters and adherence, thereby advancing the prescribability and replicability of dance interventions as a scalable public health strategy for improving sleep.

## Data Availability

The original contributions presented in the study are included in the article/Supplementary material, further inquiries can be directed to the corresponding author.
