# Supplementary material for: The effectiveness of dance interventions on sleep quality: a systematic review and meta-analysis
Source: Front Public Health. 2026 Mar 10;14:1776902. doi: 10.3389/fpubh.2026.1776902 (PMC13008660; doi:10.3389/fpubh.2026.1776902)
Supplement: Supplementary file 1 [file Data_Sheet_1.zip › R(ROB2).docx]

# Install and load the necessary packages

if (!requireNamespace("robvis", quietly = TRUE)) {

install.packages("robvis")

}

library(robvis)

if (!requireNamespace("dplyr", quietly = TRUE)) {

install.packages("dplyr")

}

library(dplyr)

library(ggplot2)

文件上传

Import- Locate the data file

# 工具列表

rob_tools()

# Summary Plots

rob_summary(data = 文献质量评价表_睡眠_20250929, tool = "ROB2", weighted = FALSE)

# 修改图表，表示总体偏倚风险判断

rob_summary(data = 文献质量评价表_睡眠_20250929, tool = "ROB2", overall = TRUE, weighted = FALSE)

# Custom color scheme

Color settings

rob_summary(data = 文献质量评价表_睡眠_20250929, tool = "ROB2", colour = "colourblind", weighted = FALSE)

把图片归纳为P

p <- rob_summary(data = 文献质量评价表_睡眠_20250929, tool = "ROB2", colour = "colourblind", weighted = FALSE)

Save path

save_path <- "C:/Users/Jason/Desktop/舞蹈-睡眠/文中图表/文献质量评价/质量评价图（睡眠）.tiff

"

*# 使用 tiff() 函数保存为 .tiff 格式，设置分辨率为 300 DPI*

tiff(save_path, width = 10, height = 8, units = "in", res = 300) *# width 和 height 以英寸为单位，分辨率设置为 300 DPI*

print(p) *# 打印图形*

dev.off() *# 关闭图形设备*

Traffic light diagram：

Generate the first step

rob_traffic_light(数据名, tool = "ROB2")

Adjust the size of the point

rob_traffic_light(数据名, tool = "ROB2", psize = 8)

Adjust colors

rob_traffic_light(data = 文献质量评价表_睡眠_20250929, tool = "ROB2",psize = 7 ,colour = c("#50589C","#636CCB","#6E8CFB"))

Classified as P

p<-rob_traffic_light(data = 文献质量评价表_睡眠_20250929, tool = "ROB2",psize = 7 ,colour = c("#50589C","#636CCB","#6E8CFB"))

closure plots

dev.off() *# 关闭图形设备*

Save path（Note the suffix.tiff

save_path <- "C:/Users/Jason/Desktop/舞蹈-睡眠/文中图表/文献质量评价/质量评价图（睡眠）.tiff

"

Tiff（）Function to save image

tiff(save_path, width = 10, height = 8, units = "in", res = 300)

print(p) *# 打印图形*

dev.off() *# 关闭图形设备*

# Chat gpt Generate code

## ===== Please install on first run ===== Installation toolkit

# install.packages(c("readxl","dplyr","robvis","ggplot2"))

library(readxl)

library(dplyr)

library(robvis)

library(ggplot2)

## ===== Read Excel（Select the most stable）=====

path_xlsx <- file.choose() # Manual selection Excel

Define the data as robdata, first page.

robdat <- read_excel(path_xlsx, sheet = 1)

## ===== Normalize column names and values ​​(but do not change the order!) =====

needed <- c("Study","D1","D2","D3","D4","D5","Overall")

robdat <- robdat[, needed]

# Standardized Low / Some concerns / High

map <- c(

"low"="Low","Low"="Low","LOW"="Low","低风险"="Low",

"some concern"="Some concerns","Some concern"="Some concerns","Some concerns"="Some concerns","某些担忧"="Some concerns",

"high"="High","High"="High","HIGH"="High","高风险"="High"

)

robdat <- robdat |>

mutate(across(c(D1,D2,D3,D4,D5,Overall), ~ {

x <- as.character(.x)

y <- ifelse(is.na(x), NA, map[ifelse(is.na(map[x]), tolower(x), x)])

ifelse(is.na(y), x, y)

}))

## Key: Locking in the order of Study factors = Excel Original order (ensuring the order remains completely unchanged)

robdat$Study <- factor(robdat$Study, levels = robdat$Study)

## ===== Traffic light diagram (default color scheme + no title)=====

p_tl <- rob_traffic_light(

data = robdat,

tool = "ROB2" # Default color scheme（Cochrane）

) + theme(plot.title = element_blank())

## Adjust the pie chart and symbols to a volume of 7–8.（7.5/5.5）

for (i in which(sapply(p_tl$layers, function(l) inherits(l$geom, "GeomPoint")))) {

p_tl$layers[[i]]$aes_params$size <- 7.5 # 改成 7 或 8 也可

p_tl$layers[[i]]$aes_params$stroke <- 0.7

}

for (i in which(sapply(p_tl$layers, function(l) inherits(l$geom, "GeomText")))) {

p_tl$layers[[i]]$aes_params$size <- 5.5

}

print(p_tl)

## ===== Bar chart showing risk percentages for each domain (default color scheme + no title; statistical chart that does not affect the order)=====

p_sum <- rob_summary(

data = robdat,

tool = "ROB2"

) + theme(plot.title = element_blank())

print(p_sum)

## ===== Export (commonly used for journals)=====

ggsave("rob2_traffic_light.png", p_tl, width = 10, height = 6, dpi = 300, bg = "white")

ggsave("rob2_traffic_light.pdf", p_tl, width = 10, height = 6, device = cairo_pdf)

ggsave("rob2_summary_bar.png", p_sum, width = 8, height = 6, dpi = 300, bg = "white")

ggsave("rob2_summary_bar.pdf", p_sum, width = 8, height = 6, device = cairo_pdf)
