# Supplementary material for: The effectiveness of dance interventions on sleep quality: a systematic review and meta-analysis
Source: Front Public Health. 2026 Mar 10;14:1776902. doi: 10.3389/fpubh.2026.1776902 (PMC13008660; doi:10.3389/fpubh.2026.1776902)
Supplement: Supplementary file 1 [file Data_Sheet_1.zip › R(Subgroup).docx]

**Find significant moderating variables**

**Strategy**

**Baseline random effects model (without moderation)**

**Four univariate mixed effects model (population, dance style, frequency, cycle) → Report QM/QMp; perform Holm multiple correction on the four p values; those still significant after correction are considered "key moderating variables"**

**For key moderating variables: Report the pooled effect and 95% CI, subgroup I², and perform post-hoc pairwise comparisons (Holm)**

**Note: For PSQI total scores on the same scale → prioritize MD (mean difference); if non-PSQI scales are mixed in the future, switch to SMD.**

# —— 0) Prepare package ----

pkgs <- c("readxl","dplyr","metafor","broom","emmeans","purrr","tidyr","ggplot2")

to_install <- setdiff(pkgs, rownames(installed.packages()))

if(length(to_install)) install.packages(to_install)

invisible(lapply(pkgs, library, character.only = TRUE))

# —— 1) Read the data & standardize column names (adjust as needed for your table)----

dat0 <- readxl::read_xlsx("/mnt/data/data.xlsx")

# Expected to contain at least：study_id, mean_t, sd_t, n_t, mean_c, sd_c, n_c, population, style, freq, duration

# If your column names are different, you can change the mapping here：

dat <- dat0 |>

rename(

study_id = study_id,

mean_t = mean_t, sd_t = sd_t, n_t = n_t,

mean_c = mean_c, sd_c = sd_c, n_c = n_c,

population = population, style = style,

freq = freq, duration = duration

) |>

mutate(across(c(population,style,freq,duration), \(x) factor(x)))

# —— 2) Calculate effect size：PSQI总分→MD ----

es <- escalc(measure = "MD",

m1i = mean_t, sd1i = sd_t, n1i = n_t,

m2i = mean_c, sd2i = sd_c, n2i = n_c,

data = dat)

# —— 3) Baseline model (unadjusted)----

fit0 <- rma(yi, vi, data = es, method = "REML")

# —— 4) Four univariate models + Holm multiple correction ----

mods <- list(

population = ~ population,

style = ~ style,

freq = ~ freq,

duration = ~ duration

)

fits <- imap(mods, ~ rma(yi, vi, mods = .x, data = es, method = "REML"))

omni_tbl <- imap_dfr(fits, ~{

tibble(

moderator = .y,

QM = .x$QM,

QMp = .x$QMp,

k = .x$k

)

}) |>

mutate(p_holm = p.adjust(QMp, method = "holm")) |>

arrange(p_holm)

# —— 5) For moderating variables of "through Holm": reported combined effect at each level + within-subgroup I² + post-hoc comparisons ----

sig_mods <- omni_tbl |> filter(p_holm < 0.05) |> pull(moderator)

get_subgroup_table <- function(var){

levs <- levels(es[[var]])

map_dfr(levs, function(L){

d <- es |> filter(.data[[var]] == L)

if(nrow(d) >= 2){

m <- rma(yi, vi, data = d, method = "REML")

tibble(Moderator = var, Level = L, K = nrow(d),

MD = as.numeric(m$b), CI_lb = m$ci.lb, CI_ub = m$ci.ub,

I2 = metafor::I2(m))

} else {

tibble(Moderator = var, Level = L, K = nrow(d),

MD = d$yi[1], CI_lb = NA_real_, CI_ub = NA_real_, I2 = NA_real_)

}

})

}

subgroup_tables <- if(length(sig_mods)) map_dfr(sig_mods, get_subgroup_table) else tibble()

# Post-hoc comparison example (using population as an example; the same applies to other variables)）

if("population" %in% sig_mods){

fit_pop <- fits$population

emm_pop <- emmeans(fit_pop, ~ population) # Sometimes it is necessary to update the emmeans and metafor versions to ensure compatibility

pairs_pop <- pairs(emm_pop, adjust = "holm")

# Check：pairs_pop

}

**Explaining heterogeneity**

**Strategies**

**A) Report baseline heterogeneity: Q/Q_p, τ², I²**

**B) Include moderating variables "through Holm" in the joint model and compare the magnitude of τ² decrease (pseudo-R²)**

**C) Stratified reporting: pooled effect across subgroups and I² within subgroups**

**D) Impact studies and leave-one-out studies to indicate whether "leverage studies" influence heterogeneity/significance (your data includes 10 studies; this is still recommended).**

# —— A) Baseline heterogeneity ----

base_hetero <- tibble(

model = "Null",

tau2 = fit0$tau2,

I2 = metafor::I2(fit0),

Q = fit0$QE,

Q_p = fit0$QEp

)

# —— B) Joint model + pseudo-R2 ----

if(length(sig_mods)){

form_joint <- as.formula(paste("~", paste(sig_mods, collapse = " + ")))

fitM <- rma(yi, vi, mods = form_joint, data = es, method = "REML")

hetero_tbl <- bind_rows(

base_hetero,

tibble(model="Moderators", tau2 = fitM$tau2, I2 = metafor::I2(fitM), Q = fitM$QE, Q_p = fitM$QEp)

) |>

mutate(pseudo_R2 = (tau2[1] - tau2)/tau2[1] * 100)

} else {

hetero_tbl <- base_hetero |> mutate(pseudo_R2 = NA_real_)

}

# —— C) Stratification results (already in subgroup_tables, with K, MD, CI, I² assigned to each significant moderating variable) ----

# —— D) Impact on diagnosis (can be assessed on a baseline model)----

inf0 <- influence(fit0) # DFBETAS / Cook's distance / hat

loo <- leave1out(fit0) # Merging effect after elimination

# Find the most impactful research (example)

idx_max <- which.max(abs(loo$estimate - as.numeric(fit0$b)))

impact_study <- es$study_id[idx_max]

**Save path**

out_dir <- "meta_outputs"

if(!dir.exists(out_dir)) dir.create(out_dir)

readr::write_csv(omni_tbl, file.path(out_dir, " Subgroups_Intergroup Differences _QM_Holm.csv"))

if(nrow(subgroup_tables)) readr::write_csv(subgroup_tables, file.path(out_dir, " Subgroups_ Merging of all levels _MD.csv"))

readr::write_csv(hetero_tbl, file.path(out_dir, " Heterogeneity_Baseline_vs_joint.csv"))
