# Supplementary material for: The effectiveness of dance interventions on sleep quality: a systematic review and meta-analysis
Source: Front Public Health. 2026 Mar 10;14:1776902. doi: 10.3389/fpubh.2026.1776902 (PMC13008660; doi:10.3389/fpubh.2026.1776902)
Supplement: Supplementary file 1 [file Data_Sheet_1.zip › Search query and screenshots of each search step.docx]

The Effectiveness of Dance Interventions on Sleep Quality：A Systematic Review and Meta-Analysis

**一、Database retrieval and retrieval strategies**

| **Datebases** | **Search strategies** |
| --- | --- |
| Web of Science | (TS=(Dancing) OR AB =(Dancing OR Dance OR Ballet OR “Jazz Dance” OR “Tap Dance” OR “Modern Dance” OR “Hip-Hop Dance” OR “Hip Hop Dance” OR “Line Dancing” OR “Salsa Dancing” OR “Square Dance”)) AND (TS=(Sleep Quality) OR AB=(“Sleep Quality” OR “sleep quality” OR “quality of sleep”)) |
| Embase | 'dancing'/exp OR dance:ti,ab,kw OR dancer:ti,ab,kw OR ballet:ti,ab,kw OR 'jazz dance':ti,ab,kw OR 'tap dance':ti,ab,kw OR 'modern dance':ti,ab,kw OR 'hip-hop dance':ti,ab,kw OR 'hip hop dance':ti,ab,kw OR 'line dancing':ti,ab,kw OR 'salsa dancing':ti,ab,kw OR 'square dance':ti,ab,kw AND 'sleep quality'/exp OR 'sleep quality':ti,ab,kw OR 'quality of sleep':ti,ab,kw |
| Pubmed | (((((((((((("Dancing"[Mesh]) OR (Dancing[Title/Abstract])) OR (Dance[Title/Abstract])) OR (Ballet[Title/Abstract])) OR (Jazz Dance[Title/Abstract])) OR (Tap Dance[Title/Abstract])) OR (Modern Dance[Title/Abstract])) OR (Hip-Hop Dance[Title/Abstract])) OR (Hip Hop Dance[Title/Abstract])) OR (Line Dancing[Title/Abstract])) OR (Salsa Dancing[Title/Abstract])) OR (Square Dance[Title/Abstract])) AND (((("Sleep Quality"[Mesh]) OR (Sleep Quality[Title/Abstract])) OR (sleep quality[Title/Abstract])) OR (quality of sleep[Title/Abstract])) |
| Cochrane | MeSH descriptor: [Dancing] explode all trees OR(Dance):ti,ab,kw OR (ballet):ti,ab,kw OR (Jazz Dance):ti,ab,kw OR (Modern Dance):ti,ab,kw OR (Tap Dance):ti,ab,kw OR(Hip-Hop Dance):ti,ab,kw OR (Hip Hop Dance):ti,ab,kw OR (Line Dancing):ti,ab,kw OR (Salsa Dancing):ti,ab,kw OR (Square Dance):ti,ab,kw AND MeSH descriptor: [Sleep Quality] explode all trees OR(sleep quality):ti,ab,kw OR (quality of sleep):ti,ab,kw |

**二、Search screenshot**

2.1 Web of Science , Search time：20250923,171 articles


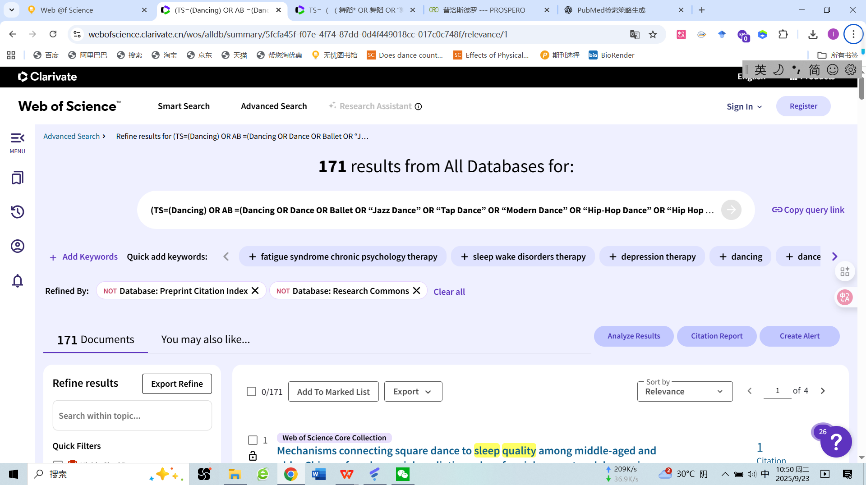


2.2 Embase ,Search time：20250923,107 articles


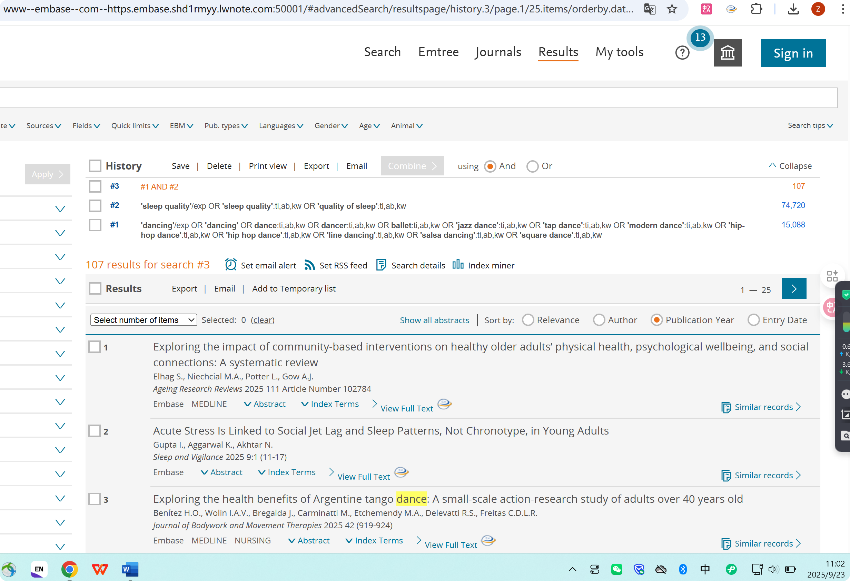


2.3 Pubmed , Search time：20250923, 44 articles


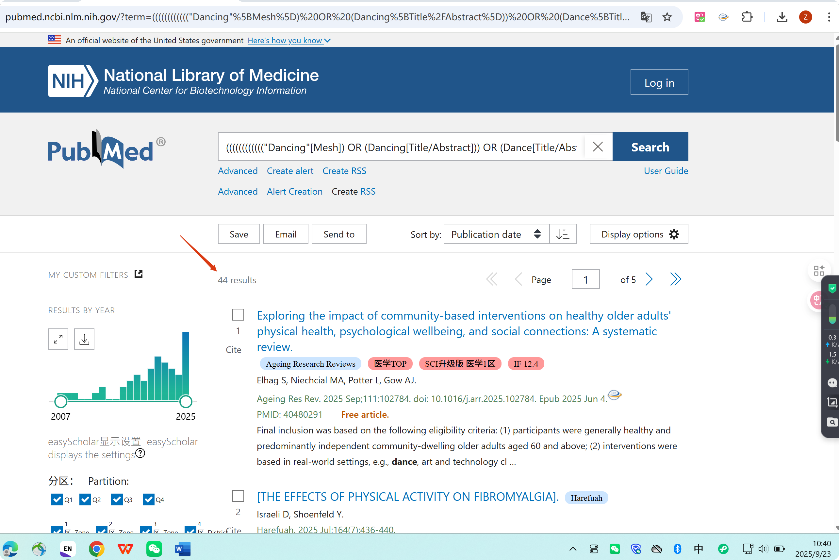


2.4 Cochrane , Search time：20250923, 57 articles


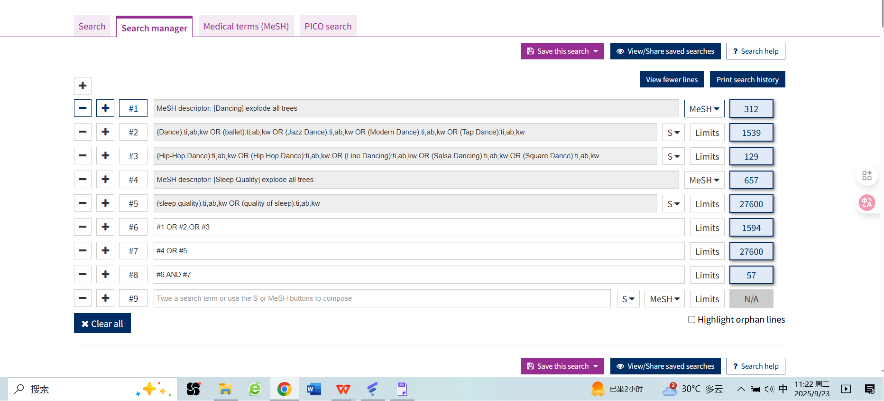


**三、Screening strategy and flowchart (illustrated)**

**Identification of studies via databases and registers**

**Identification**

Records removed *before screening*:

Duplicate records removed (n = 175)

Records identified from:

(n = 379)

Web of Science (n = 171)

Embase (n = 107)

PubMed (n = 44)

Cochrane (n = 57)

Records excluded based on title or abstract (n = 183)

Records screened

(n = 204)

**Screening**

Reports excluded based on full text(n = 11)

Following conditions not met:
Intervention (n = 2)
Outcome (n = 5)
Study design (n = 4)

Reports assessed for eligibility

(n = 21)

Studies included in review

(n = 10)

**Included**

***Consider, if feasible to do so, reporting the number of records identified from each database or register searched (rather than the total number across all databases/registers).**

****If automation tools were used, indicate how many records were excluded by a human and how many were excluded by automation tools.**
