# Supplementary figures and images for: The effectiveness of dance interventions on sleep quality: a systematic review and meta-analysis
Source: Front Public Health. 2026 Mar 10;14:1776902. doi: 10.3389/fpubh.2026.1776902 (PMC13008660; doi:10.3389/fpubh.2026.1776902)

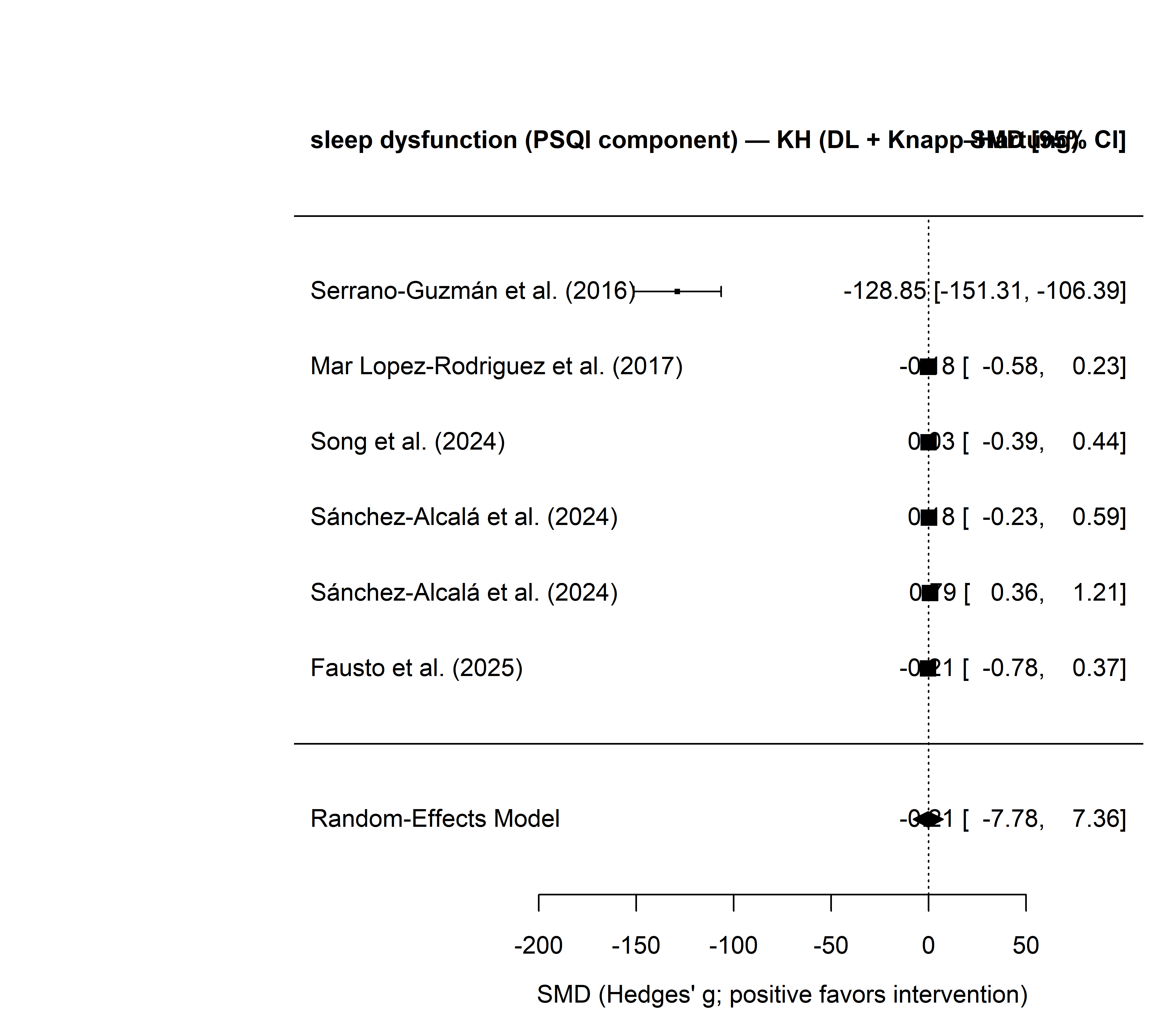

Supplement: Supplementary file 1 [file Data_Sheet_1.zip › Domains effect of PSQI/Daytime dysfunction/Daytime dysfunction_Forest plot_KH_600dpi.png]

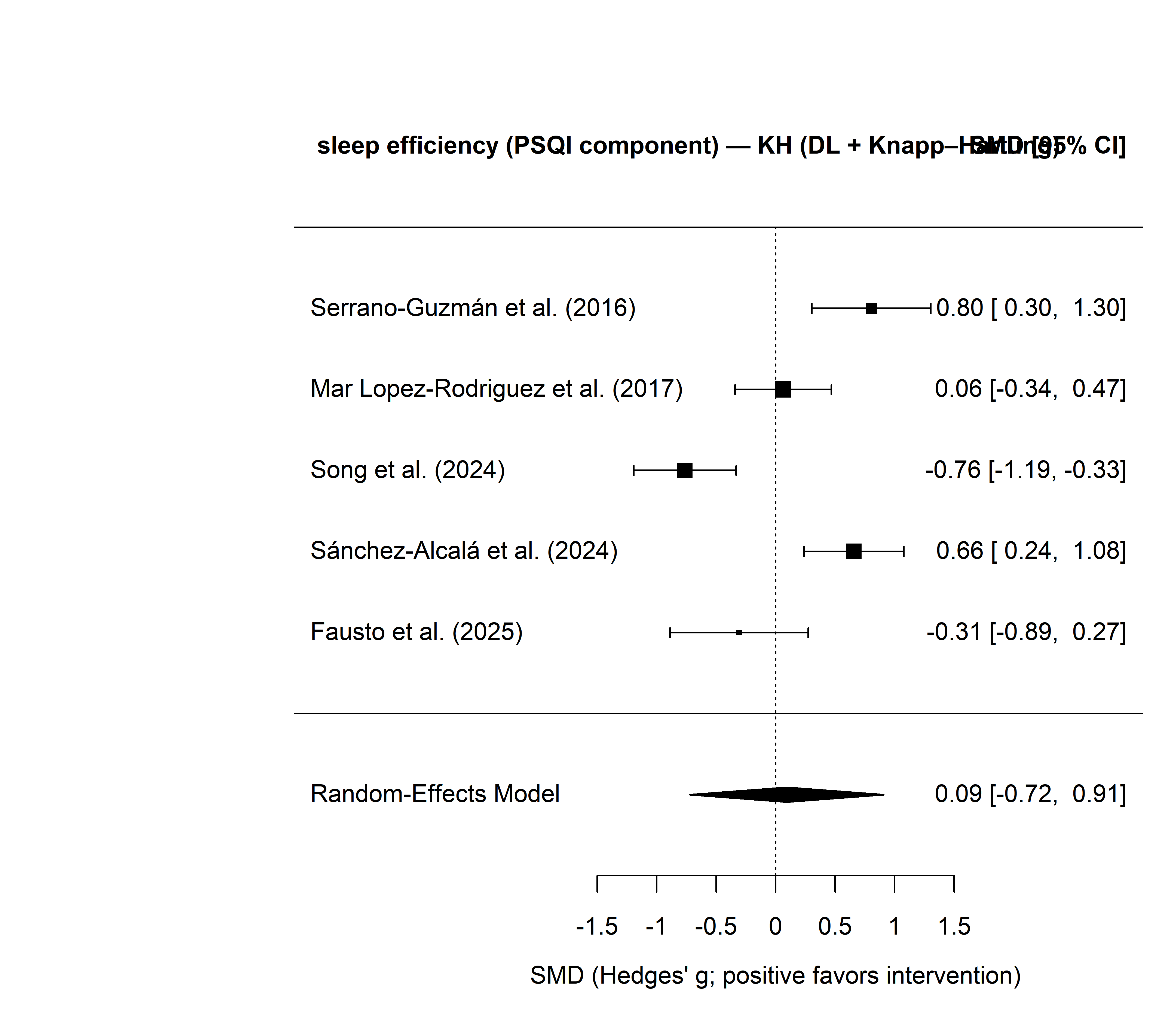

Supplement: Supplementary file 1 [file Data_Sheet_1.zip › Domains effect of PSQI/Habitual sleep efficiency/Habitual sleep efficiency_Forest plot_KH_600dpi.png]

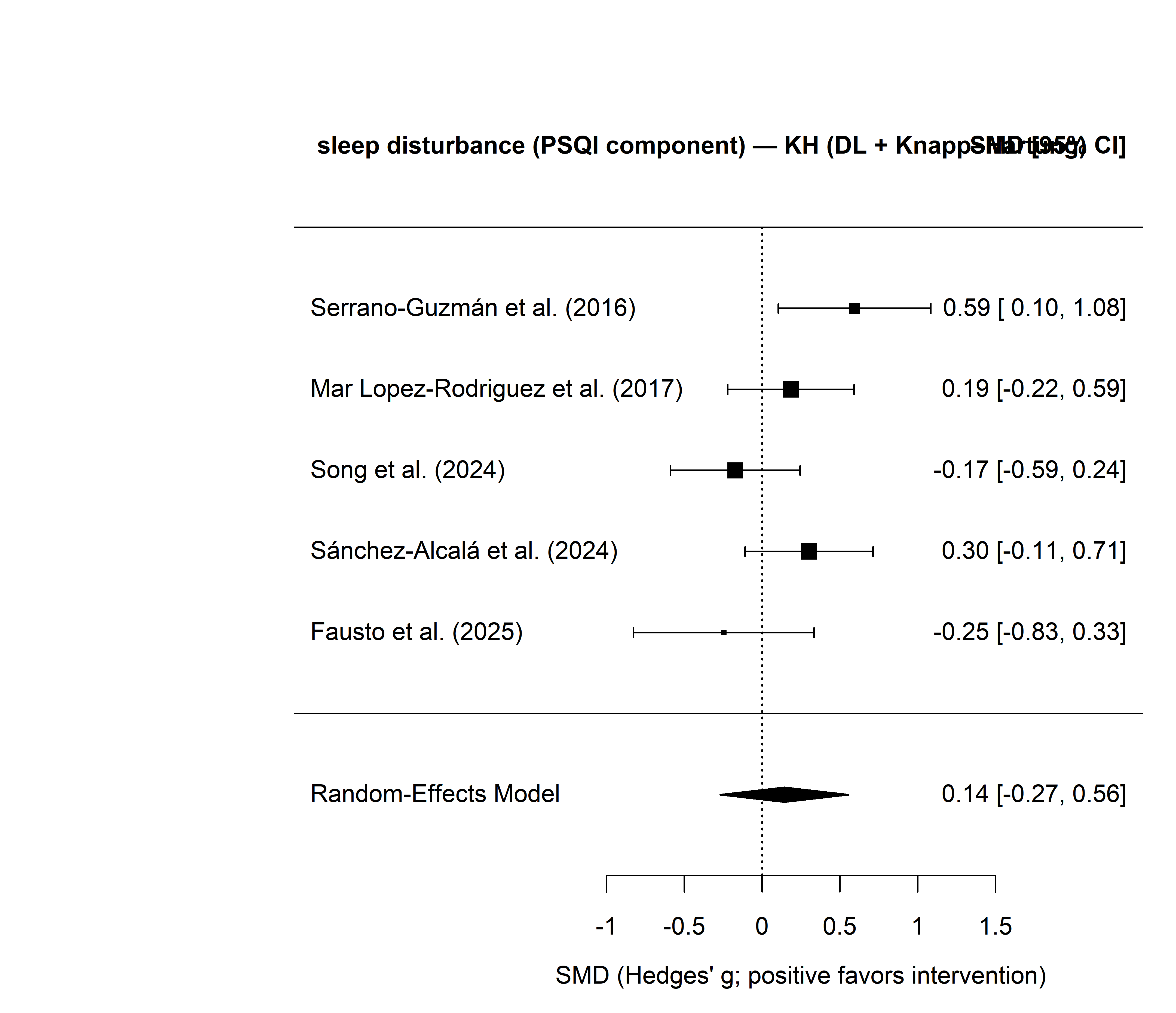

Supplement: Supplementary file 1 [file Data_Sheet_1.zip › Domains effect of PSQI/Sleep disturbances/Sleep disturbances_Forest plot_KH_600dpi.png]

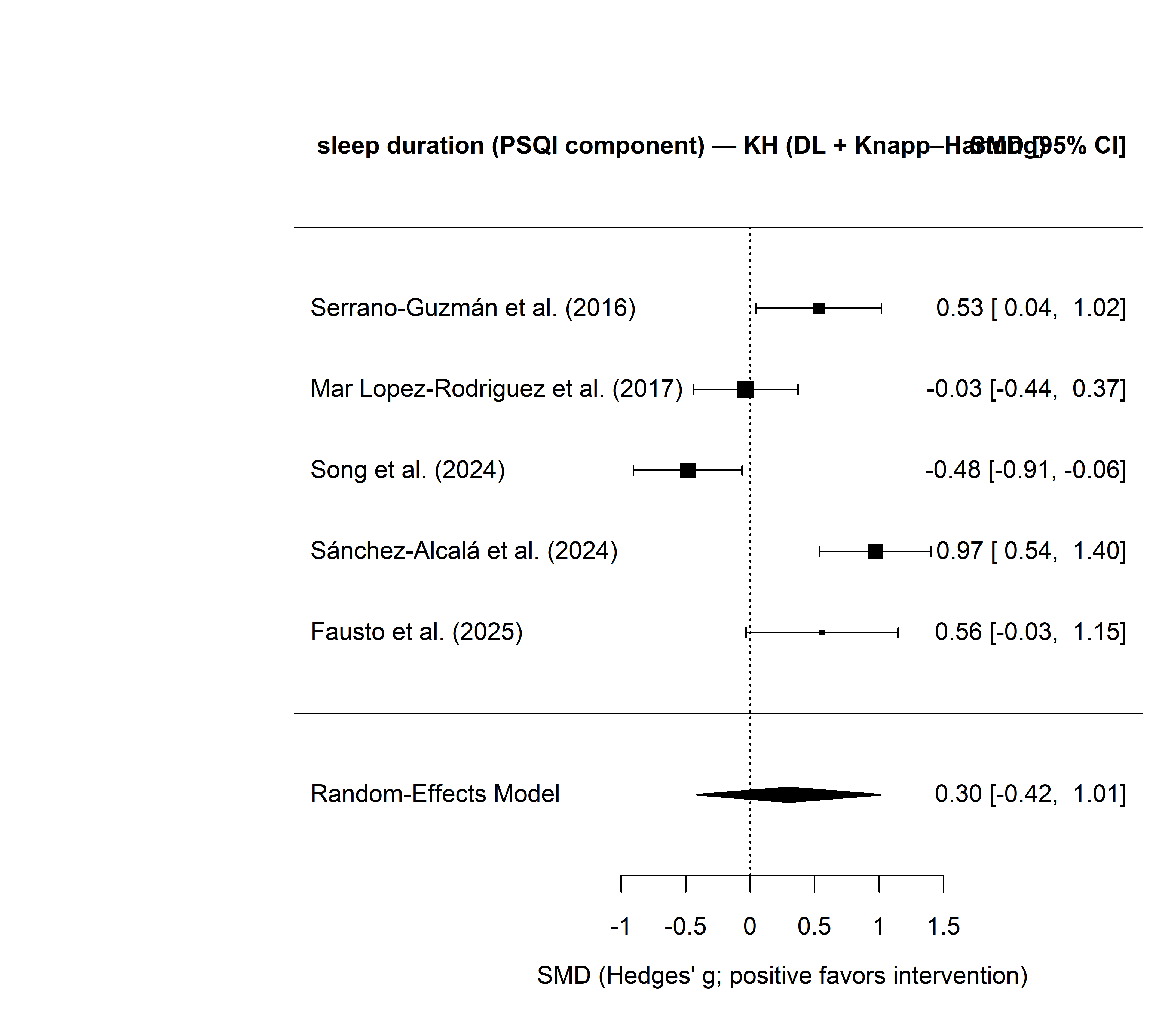

Supplement: Supplementary file 1 [file Data_Sheet_1.zip › Domains effect of PSQI/Sleep duration/Sleep duration_Forest plot_KH_600dpi.png]

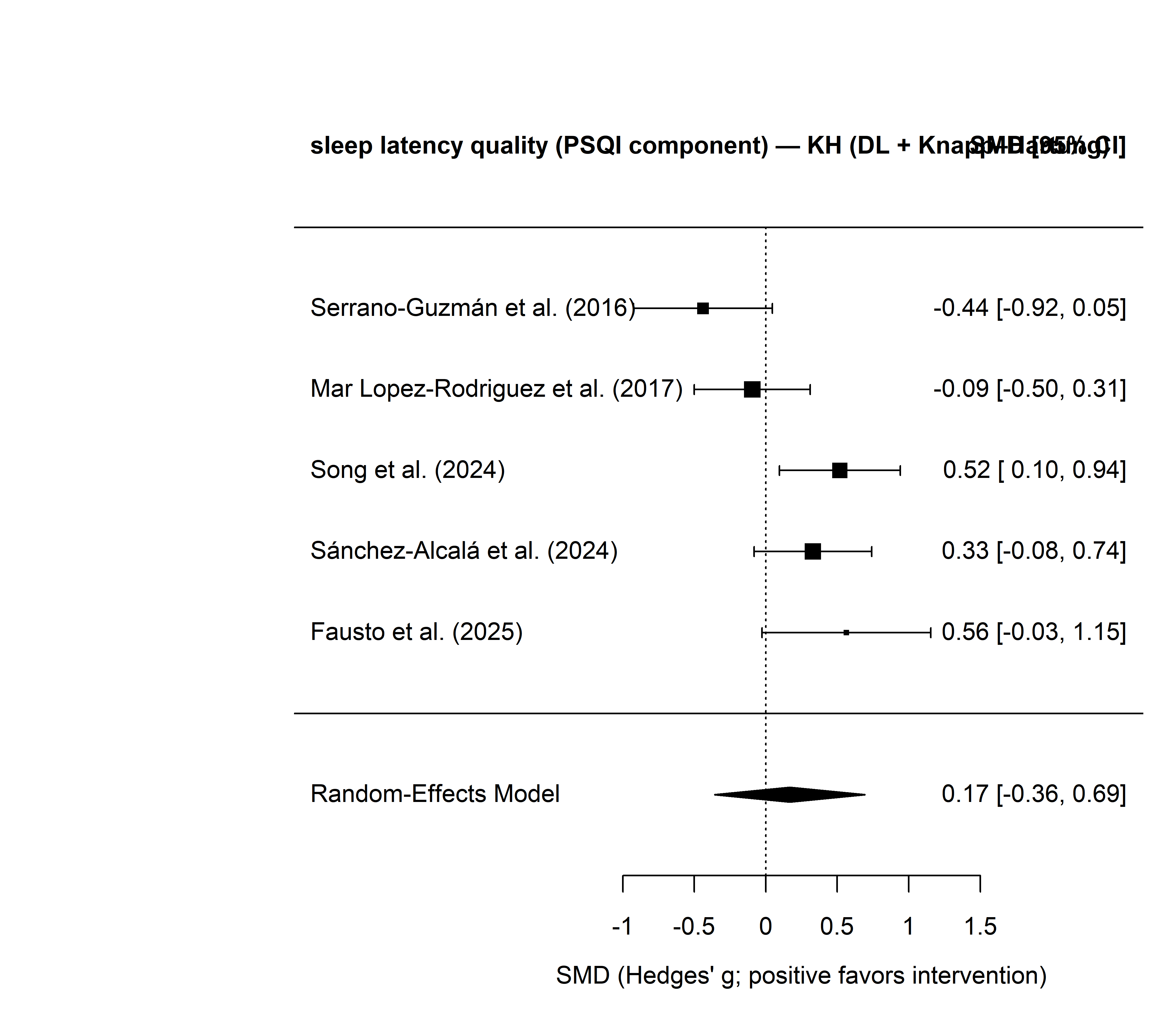

Supplement: Supplementary file 1 [file Data_Sheet_1.zip › Domains effect of PSQI/Sleep latency/Sleep latency_Forest plot_KH_600dpi.png]

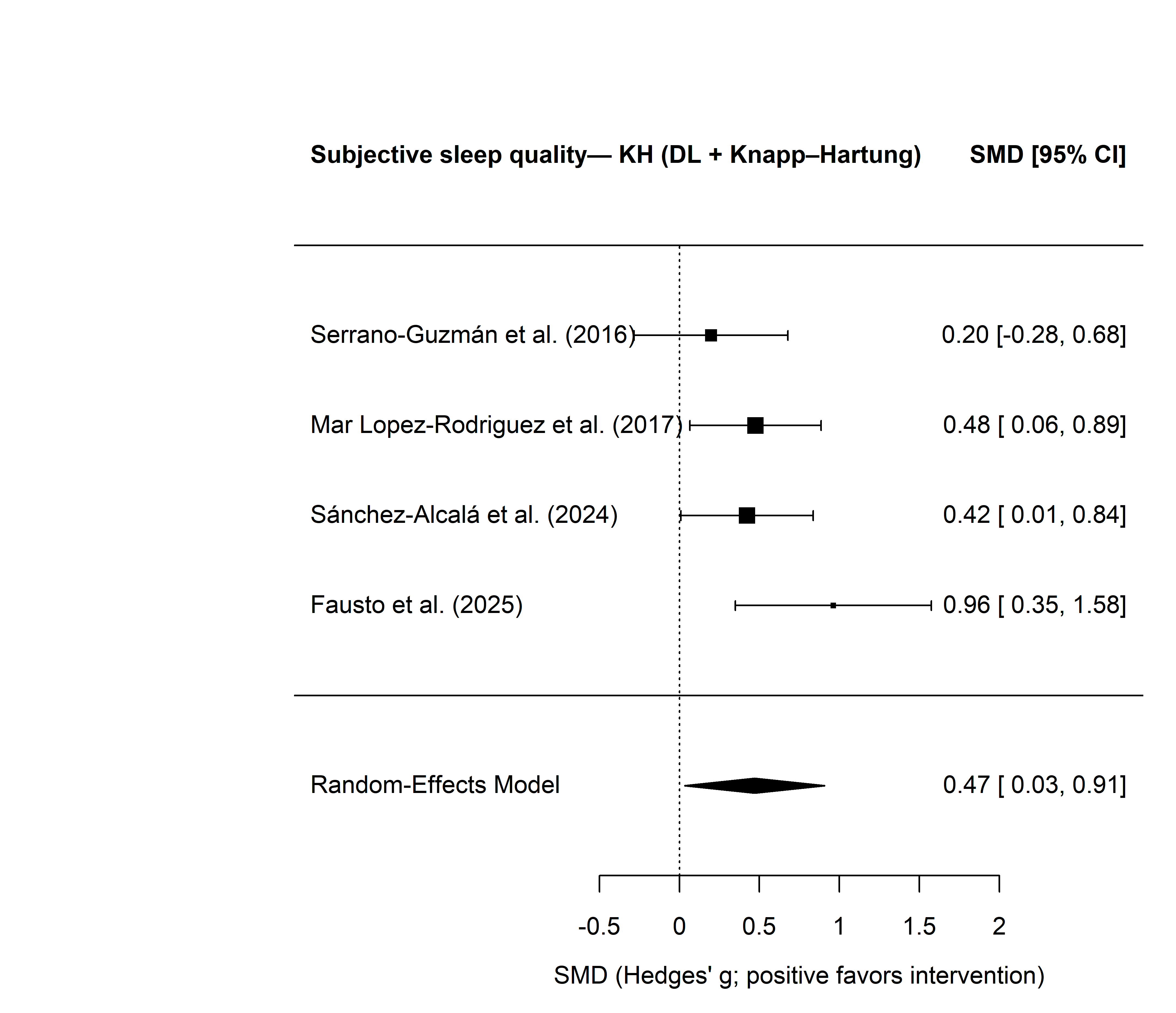

Supplement: Supplementary file 1 [file Data_Sheet_1.zip › Domains effect of PSQI/Subjective sleep quality/Subjective sleep quality_Forest plot_KH_600dpi.png]

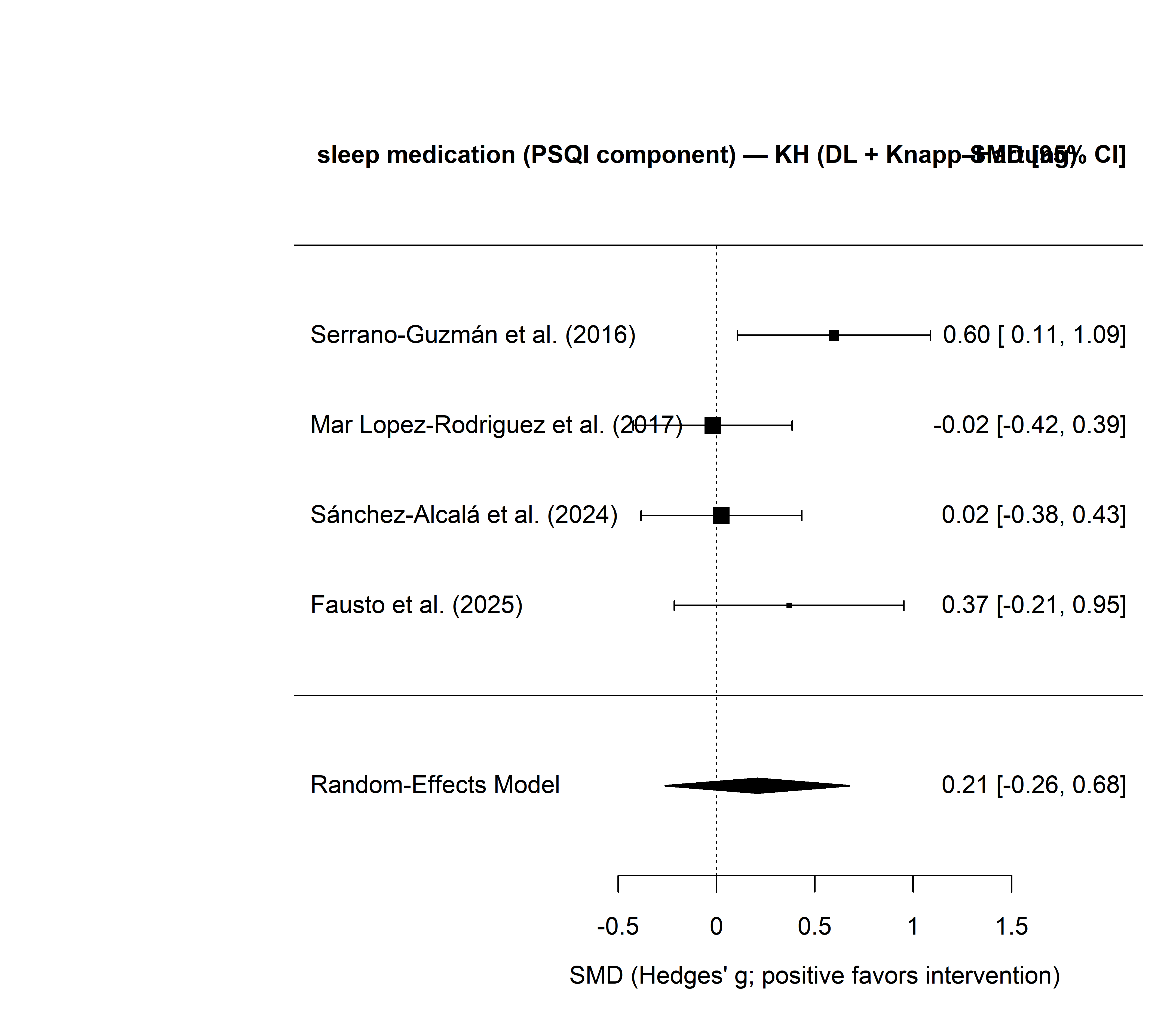

Supplement: Supplementary file 1 [file Data_Sheet_1.zip › Domains effect of PSQI/Use of sleeping medication/Use of sleeping medication_Forest plot_KH_600dpi.png]

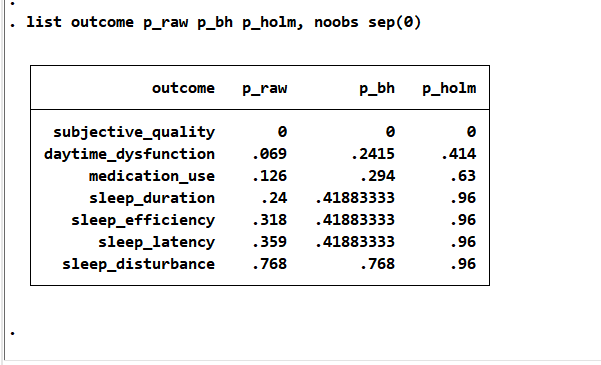

Supplement: Supplementary file 1 [file Data_Sheet_1.zip › holm.jpg]
